# Comparison of IVF/ICSI outcomes in advanced reproductive age patients with polycystic ovary syndrome and advanced reproductive age normal controls: a retrospective cohort study

**DOI:** 10.1186/s12884-023-05732-0

**Published:** 2023-06-14

**Authors:** Xing Zhang, Fang Lian, Danqi Liu

**Affiliations:** 1 Shandong University of Traditional Chinese Medicine, Jinan, China; 2 Affiliated Hospital of Shandong University of Traditional Chinese Medicine, Jinan, China

**Keywords:** Advanced reproductive age, Polycystic ovary syndrome, IVF/ICSI, Clinical pregnancy rate, Live birth rate

## Abstract

**Background:**

At present, there are few studies on whether there is reproductive advantage in advanced polycystic ovary syndrome (PCOS) patients, and the existing research results are also controversial. Some research results show that the reproductive window of advanced reproductive age patients with polycystic ovary syndrome is longer than that of the normal control group, and the clinical pregnancy rate and cumulative live birth rate of in vitro fertilization / intracytoplasmic sperm injection(IVF/ICSI)are higher. However, some studies have contradicted the results, and believed that the clinical pregnancy rate and cumulative live birth rate in IVF/ICSI in advanced PCOS patients and normal control groups were roughly similar. This retrospective study aimed to compare IVF/ICSI outcomes in advanced reproductive age patients with PCOS and in advanced reproductive age patients with tubal factor infertility alone.

**Methods:**

A retrospective analysis was performed on advanced reproductive age (age ≥ 35 years) patients who received their first IVF/ICSI cycle between January 1, 2018 and December 31, 2020. This study was divided into two groups, one group was PCOS group, the other group was control group, namely tubal factor infertility group, a total of 312 patients and 462 cycles were enrolled. Compare the differences in outcomes such as cumulative live birth rate and clinical pregnancy rate between the two groups.

**Results:**

In fresh embryo transfer cycles(ET), there was no statistically significant difference in live birth rate [19/62 (30.6%) vs. 34/117 (29.1%), *P* = 0.825] and clinical pregnancy rate [24/62 (38.7%) vs. 43/117 (36.8%), *P* = 0.797] between the PCOS and control groups.In the frozen embryo transfer (FET) cycle, the difference in cumulative live birth rate [63/217 (29.0%) vs. 14/66 (21.2%), *P* = 0.211] and clinical pregnancy rate [74/217 (34.1%) vs. 18/66 (27.3%), *P* = 0.300] were not statistically significant between the two groups.

**Conclusions:**

The IVF/ICSI outcomes of advanced reproductive age patients with PCOS are similar to those of advanced reproductive age patients with tubal factor infertility alone, and the clinical pregnancy rate and live birth rate are roughly the same. Age is an important factor that affects clinical pregnancy rate. It is recommended that patients with PCOS complicated by infertility seek medical treatment as soon as possible to obtain better pregnancy outcomes.

**Supplementary Information:**

The online version contains supplementary material available at 10.1186/s12884-023-05732-0.

## Background

Polycystic ovary syndrome (PCOS) is a common endocrine disorder in women of childbearing age, and about 6–10% of women of childbearing age are affected by the disease to varying degrees [1, 2]. Among them, infertility caused by abnormal ovulation is deeply troubled by many women with reproductive requirements. Studies have shown that anovulation is attributable to approximately 30% of couples seeking treatment for infertility [3], and that 80 to 90% of anovulatory patients are PCOS patients [4, 5]. Anovulation is one of the common manifestations in patients with PCOS, and other pathophysiological changes of PCOS, such as obesity, high androgen levels, and metabolic abnormalities, aggravate the complexity of infertility problems in patients from various aspects [6–8]. Advanced age is even worse for infertile patients. Given the significant advantage of ovarian reserve in patients with polycystic ovary syndrome [9–11], some studies have suggested that women with polycystic ovary syndrome at an advanced age have a longer reproductive window and greater reproductive potential, but the determinants of a woman’s reproductive potential include a variety of factors, including embryonic, endometrial and pregnancy complications, in addition to oocyte factors [12–15]. Therefore, it remains controversial whether women with polycystic ovary syndrome at an advanced age have a greater reproductive advantage [16, 17]. This study was a retrospective analysis of advanced reproductive age patients treated with ART at our center to compare in vitro fertilization / intra cytoplasmic sperm injection(IVF/ICSI)outcomes in advanced reproductive age patients with PCOS and those with isolated tubal factor infertility.

## Materials and methods

### Study design and participants

Advanced reproductive age patients (age ≥ 35 years old) who received IVF/ICSI cycles for the first time between January 1, 2015 and December 31, 2018 were screened for retrospective identification in the database of the Reproductive Center of the Affiliated Hospital of Shandong University of Traditional Chinese Medicine. A total of 156 patients with PCOS and 344 patients with infertility with only tubal factor were included. According to the cause of infertility, the patients were divided into two groups, one group was PCOS group, and the other group was control group, namely tubal factor infertility group. Inclusion criterions: (1) The diagnostic criteria for PCOS are based on the Rotterdam Consensus published in 2003 [18]: Oligonovulation or anovulation; clinical or biochemical signs of hyperandrogenism; polycystic ovary. PCOS can be diagnosed when two of the above three conditions are met, and other causes such as congenital adrenal hyperplasia, androgen-secreting tumor, and Cushing’s syndrome are excluded; (2) In the control group, the indications for assisted pregnancy were advanced age and tubal factors; in the PCOS group, the indications for assisted pregnancy were advanced age and ovulation disorders; in both groups, the male partner had normal reproductive function.3. The age of the patient is ≥ 35 years old. Exclusion Criterions: (1) No embryos available; (2) Infertile patients with uterine submucosal fibroids, endometriosis, uterine malformations or other genital tumors; (3) History of ovarian surgery in the past; (4) Suffering from other endocrine diseases. Assess and record patient characteristics, including age, body mass index (BMI), type of infertility, years of infertility, number of oocytes retrieved, number of normal fertilization, number of available embryos, number of high-quality embryos, number of embryos transferred, and clinical pregnancy rate and live birth rate. There was a statistically significant difference in age between the initial control group (n = 344) and the PCOS group (n = 156) [median age in the control group 39.0 (interquartile range 37.0–42.0; PCOS 37.0 (36.0–39.0), *z*=-5.557, *P* < 0.001)]. To ensure the scientificity and credibility of the results, we adopted the PSM method, using nearest-neighbor random matching according to the patient’s age, and matched at a 1:1 ratio. This study was approved by the Ethics Committee of the Affiliated Hospital of Shandong University of Traditional Chinese Medicine.

### Controlled ovarian stimulation (COS) regimen

COS regimens include gonadotropin-releasing hormone agonist (GnRH-a) regimens and gonadotropin-releasing hormone antagonist (GnRH-ant) regimens, the GnRH-a scheme includes GnRH-a long scheme (short-acting GnRH-a long scheme and long-acting GnRH-a scheme), GnRH-a short scheme, and GnRH-a ultra-short scheme. Physicians choose the appropriate plan according to the actual situation of the patient.

### GnRH-ant regimen

Blood hormones were measured on the third day of menstruation, including basal serum follicle-stimulating hormone (FSH), luteinizing hormone (LH), estradiol (E_2_), progesterone (P), and vaginal ultrasound to evaluate the basic status of ovaries and follicles, gonadotropin (Gn) can start the controlled ovarian stimulation program on the same day for those who can enter the cycle.Gn includes recombinant follicle-stimulating hormone, human menopausal gonadotropin, and recombinant human luteinizing hormone for injection. The choice of Gn type and dose is determined according to the patient’s endocrine hormone level, antral follicle count (AFC), BMI, etc. Close monitoring of follicular development and blood hormone changes in patients,, when the dominant follicle diameter is ≥ 12 mm and the serum E_2_ level is greater than 300pg/ml, 0.25 mg/day of cetrelix acetate for injection is administered until the trigger day. When at least one follicle is ≥ 18 mm in diameter or 3 follicles are ≥ 17 mm in diameter, 4000-10000IU human chorionic gonadotropin (hCG) or 250ug recombinant human chorionic gonadotropin is given to induce follicle maturation. The oocytes were retrieved 36 h after injection.

### GnRH-a long scheme (short-acting GnRH-a long scheme and long-acting GnRH-a scheme)

One-time injection of long-acting GnRH-a preparation in mid-luteal phase or daily injection of short-acting GnRH-a preparation until the day of HCG injection. Pituitary down regulation was assessed by measuring blood FSH, LH, E_2_ levels and transvaginal ultrasound 14–21 days after injection,, When the pituitary reaches the following down regulation standards (down regulation standard: LH < 5 ~ 10IU/L, E_2_ < 50pg/ml, intima < 4 ~ 5 mm, no functional cyst), start to use exogenous gonadotropin to stimulate ovulation, until HCG day. The dose of the drug is determined according to the patient’s BMI, endocrine level, and follicle count. When at least one follicle was ≥ 18 mm in diameter or 3 follicles were ≥ 17 mm in diameter, 4000-10000IU hCG or 250ug recombinant human chorionic gonadotropin was administered to induce follicle maturation. The oocytes were retrieved 36 h after injection.

### GnRH-a short scheme

Short-acting GnRH-a preparations were started on the second day of the menstrual cycle, and Gn was used to induce ovulation on the third day until HCG day. The dose of the drug is determined according to the patient’s BMI, endocrine level, and follicle count. HCG was injected when the dominant follicle diameter was greater than or equal to 18 mm, and the oocytes were taken out 36 h after injection.

### GnRH-a ultra-short scheme

Short-acting GnRH-a preparations were started on the second day of the menstrual cycle, and Gn was used to induce ovulation on the third day until HCG day. Short-acting GnRH-a formulations were discontinued on day 4 of Gn use. HCG was injected when the dominant follicle diameter was greater than or equal to 18 mm, and the oocytes were taken out 36 h after injection.

### Embryo scoring and embryo transfer

For cleavage stage embryos, their quality was graded using Cummins criteria [19]. The number of oocytes retrieved is the number of oocytes obtained on the day of oocyte retrieval; the number of normal fertilization refers to the number of cells with two pronuclei observed under the microscope after fertilization; The number of embryos is the total number of embryos finally formed; the number of available embryos = the number of embryos of grade I + the number of embryos of grade II + the number of embryos of grade III; the number of high-quality embryos = the number of embryos of grade I + the number of embryos of grade II.

In a fresh transfer (ET) cycle, embryo transfer is performed 3 to 5 days after egg retrieval, with a maximum of 3 embryos transferred. For patients with potential for fresh embryo transfer, luteal support, progesterone injection or progesterone gel vaginally, is given from the day of oocyte retrieval until a pregnancy test is performed. The dose of the drug is determined according to the patient’s BMI, endocrine level, and follicle count. For patients who do not have a satisfactory pregnancy outcome with ET and for patients who are not suitable for a fresh transfer cycle, such as the patient with ovarian hyperstimulation syndrome (OHSS) or is at risk of OHSS, the embryos will be frozen for an elective frozen cycle transfer (FET). The patients in the FET group consisted of three parts: the first part was patients who had undergone ET but had an unsatisfactory ET result; the second part was patients who were not suitable for EF and had to FET; and the third part was patients who had continued FET after the first FET and did not have a satisfactory result. In summary, the ET and FET groups are not in a complete exclusion relationship and the live birth rate in the FET group is the cumulative live birth rate. In the FET cycle, according to the actual situation of the patient, the natural cycle, the artificial cycle or the alternative cycle is used for endometrial preparation. Embryo transfer is then carried out 3 to 5 days after ovulation, with a maximum of 3 embryos transferred.

### Outcome measure

The primary outcome of this study was live birth rate, and secondary outcomes included clinical pregnancy rate, number of high-quality embryos, number of available embryos, and number of oocytes retrieved. The serum HCG level of the patient was detected 14 days after transplantation, if serum HCG > 10 IU/L, it indicated a positive pregnancy test. Ultrasound was performed 28 days after embryo transfer, and a clinical pregnancy was diagnosed if a gestational sac was detected on the ultrasound. Live birth refers to one of the four life phenomena of heartbeat, respiration, umbilical cord pulsation and voluntary muscle movement at birth, including full-term and premature infants.

### Statistical analysis

All data were statistically analyzed by SPSS 23.0 software, and *P* < 0.05 indicated that the difference was statistically significant. When comparing the measurement data between the two groups, if the data conformed to normality and homogeneity of variance, two independent samples *t* test was used, and it was expressed as mean ± standard deviation (‾*x ± s* ), if not, the Wilcoxon rank sum test of two independent samples is used, and the median (M), 25% quantile (Q25), and 75% quantile (Q75) are used to indicate; the chi-square test was used to compare the rates between the two groups; modified poisson regression for live birth.

## Results

### Basic characteristics of the patient

After 1:1 matching using the PSM method, the final control group and PCOS group included 156 patients each, the age of the patients in the control group was 37.0 (35.0–39.0) years, and the age of the patients in the PCOS group was 37.0 (36.0–39.0) years.There was no significant difference in the overall age distribution between the two groups (*z*=-0.009, *P* = 0.993), and subsequent comparisons were possible. The basic characteristics of the patients are detailed in Table 1.

The BMI of the patients in the control group was (22.30 ± 2.31) kg/m^2^, and the 95% confidence interval was 21.94–22.67; BMI of patients in PCOS group was (25.52 ± 3.38) kg/m^2^, 95% confidence interval was 24.98–26.05;there was a statistically significant difference in the overall mean of body weight between the two groups (*t*=-9.810, *P* < 0.001).

In the control group, there were 42/156 (26.9%) patients with primary infertility and 114 /156(73.1%) patients with secondary infertility; in the PCOS group, there were 78/156 (50%) patients with primary infertility and 78/156 (50%) patients with secondary infertility; the difference of infertility types between the two groups was statistically significant (X^2^ = 17.550, *P* < 0.001).

The years of infertility in the control group were 4.0 (2.0–6.0) years, and the infertility years in the PCOS group was 4.0 (3.00-7.75) years, the difference in the overall infertility years between the two groups was not statistically significant (*z*=-1.916, *P* = 0.055).

**Table 1:** Baseline characteristics of patients

Variables	Control	PCOS	*P* value
Number of patients	156	156	
Age of woman (year)	37.0(35.0–39.0)	37.0(36.0–39.0)	0.993
BMI (kg/m^2^)	22.30 ± 2.31	25.52 ± 3.38	*P* < 0.001
Infertility duration(year)	4.0(2.0–6.0)	4.0(3.00-7.75)	0.055
Type of infertility			*P* < 0.001
Primary infertility	42/156(26.9%)	78/156(50%)	
Secondary infertility	114/156(73.1%)	78/156(50%)	

BMI: Body Mass Index. The denominator is the total number of participants in each group. Values are described as median (25th quartile,75th quartile) or number/total number ( percentage ) or *mean ± SD*. All *P* values were assessed with the use of student’s t-test or *χ*^*2*^

### Cycle characteristics

The cycle characteristics of patients are shown in Table 2. A total of 462 cycles were finally included, including 183 cycles in the control group, including 117/183 (63.9%) cycles of ET and 66/183 (36.1%) cycles of FET; PCOS group had a total of 279 cycles, including 62/279 (22.2%) ET cycles and 217/279 (77.8%) FET cycles; there was a statistically significant difference in the overall cycle type between the two groups (*X*^*2*^ = 81.018, *P* < 0.001).

The number of oocytes retrieved in the control group was 9.0 (4.0–13.0), and the number of oocytes retrieved in the PCOS group was 17.0 (11.25–22.75), there was a statistically significant difference in the number of oocytes retrieved between the two groups (*z*=-8.985, *P* < 0.001).

The number of normal fertilizations in the control group was 5.0 (3.0–9.0), and the number of normal fertilizations in the PCOS group was 10.0 (6.0–14.0), and there was a statistically significant difference in the number of normal fertilization between the two groups (*z*=-7.285, *P* < 0.001).

The number of available embryos in the control group was 4.0 (2.0–6.0), and the number of available embryos in the PCOS group was 7.0 (4.0–11.0), there was a statistically significant difference in the number of available embryos between the two groups (*z*=-5.895, *P* < 0.001).

The number of high-quality embryos in the control group was 3.0 (1.0–5.0), and the number of high-quality embryos in the PCOS group was 4.0 (2.00-7.75), and there was a statistically significant difference in the number of high-quality embryos between the two groups (*z*=-4.291, *P* < 0.001).

In ET cycle, the number of embryos transferred in the control group was 2.0 (1.0–2.0), of which 34/117 (29.1%) transferred 1 embryo, 64/117 (54.7%) transferred 2/117 embryos, and 19/117 (16.2%) transferred 3 embryos; the number of embryos transferred in the PCOS group was 2.0 (2.0–2.0), of which 6/62 (9.7%) were transferred with 1 embryo, 46/62 (74.2%) with 2 embryos, and 10/62 (16.1%) with 3 embryos, there was a statistically significant difference in the number of embryos transferred between the two groups (*z*=-2.048, *P* = 0.041).

In FET cycle, the number of embryos transferred in the control group was 2.0 (2.0–2.0), of which 14/66 (21.2%) transferred 1 embryo, 43/66 (65.2%) transferred 2 embryos, and 9/66 ( 13.6%) transfer 3 embryos; the number of embryos transferred in the PCOS group was 2.0 (2.0–2.0), with 31/217 (14.3%) transferring 1 embryo, 140/217 (64.5%) transferring 2 embryos, and 46/217(21.2%) transferring 3 embryos, there was no significant difference in the number of embryos transferred between the two groups (*z*=-1.733, *P* = 0.083).

**Table 2:** IVF/ICSI cycle characteristics between groups

Variables	Control	PCOS	*P* value
Type of cycle			*P* < 0.001
ET	117/183(63.9%)	62/279(22.2%)	
FET	66/183(36.1%)	217/279(77.8%)	
Number of oocytes retrieved	9.0(4.0–13.0)	17.0(11.25–22.75)	*P < 0.001*
Number of normal fertilizations	5.0(3.0–9.0)	10.0(6.0–14.0)	*P < 0.001*
Number of available embryos	4.0(2.0–6.0)	7.0(4.0–11.0)	*P < 0.001*
Number of high-quality embryos	3.0(1.0–5.0)	4.0(2.00-7.75)	*P < 0.001*
ET			
Average number of embryos transferred, n	2.0(1.0–2.0)	2.0(2.0–2.0)	0.041
with 1 embryo transferred	34/117(29.1%)	6/62(9.7%)	
with 2 embryos transferred	64/117(54.7%)	46/62(74.2%)	
with 3 embryos transferred	19/117(16.2%)	10/62(16.1%)	
FET			
Average number of embryos transferred, n	2.0(2.0–2.0)	2.0(2.0–2.0)	0.083
with 1 embryo transferred	14/66(21.2%)	31/217(14.3%)	
with 2 embryos transferred	43/66(65.2%)	140/217(64.5%)	
with 3 embryos transferred	9/66(13.6%)	46/217(21.2%)	

Values are described as median (25th quartile,75th quartile) or number/total number (percentage). The denominator of the cycle type in each group was the total number of cycles, which included the number of ET cycles and the number of FET cycles. The denominator of ET cycle in the number of transferred embryos is both the number of patients with fresh embryo transfer in each group and the number of ET cycles. The denominator of FET cycles in the number of transplanted embryos was the total number of FET cycles in each group

### Pregnancy outcomes

Pregnancy outcomes between the groups are shown in Tables 3, 4 and 5. During the ET cycle, the clinical pregnancy rate was 43/117 (36.8%) in the control group, 24/62 (38.7%) in the PCOS group, and there was no significant difference in the overall clinical pregnancy rate between the two groups (*X*^*2*^ = 0.066, *P* = 0.797). The live birth rate (including full-term birth and preterm birth) in the control group was 34/117(29.1%), specific pregnancy outcomes including 27/43(62.8%) full-term births, 7/43(16.3%)preterm births, and 1/43(2.3%) ectopic pregnancy, 8/43(18.6%) miscarriage; the live birth rate in the PCOS group was 19/62 (30.6%), specific pregnancy outcomes including 13/24(54.2%) full-term births, 6/24(25.0%) premature births, 2/24(8.3%) ectopic pregnancy, and 3/24(12.5%) miscarriage,there was no statistically significant difference in the overall live birth rate between the two groups (*X*^*2*^ = 0.049, *P* = 0.825). Age, type of infertility, infertility duration, BMI, number of oocytes retrieved, number of normal fertilisations, number of available embryos, number of high-quality embryos and live birth rate were included in a modified poisson regression to investigate the effect of each factor on the live birth rate. The results showed a significant effect of age on live birth rate in the control group (RR = 0.864, 95% IC 0.761–0.980, *P* = 0.023) and no factors could have a significant effect on live birth rate in the PCOS group (*P* > 0.05).

In the FET cycle, the clinical pregnancy rate of the control group was 18/66(27.3%), the clinical pregnancy rate of the PCOS group was 74/217(34.1%), and there was no significant difference in the overall clinical pregnancy rate between the two groups (*X*^*2*^ = 1.076, *P* = 0.300).The cumulative live birth rate (including full-term birth and preterm birth) in the control group was 14/66(21.2%), specific pregnancy outcomes including 11/18(61.1%) full-term births, 3/18(16.7%) preterm births, and 1/18(5.6%) ectopic pregnancy. ), 3/18(16.7%) miscarriage; the cumulative live birth rate in the PCOS group was 63/217(29.0%), including 50/74(67.6%) full-term births, 13/74(17.6%) premature births, 1/74(1.4%) ectopic pregnancy, and 10/74(13.5%) miscarriage,there was no statistically significant difference in the overall cumulative live birth rate between the two groups (*X*^*2*^ = 1.563, *P* = 0.211). Age, type of infertility, infertility duration, BMI, number of oocytes retrieved, number of normal fertilisations, number of available embryos, number of high-quality embryos and live birth rate were included in a modified poisson regression to investigate the effect of each factor on the live birth rate. The results showed that age and infertility duration could have a significant effect on live birth rate in the control group (age: RR = 0.714, 95% IC 0.522–0.975, *P* = 0.034; infertility duration: RR = 1.181, 95% IC 1.007–1.384, *P* = 0.041) and no factor could have a significant effect on live birth rate in the PCOS group (*P* > 0.05) .

Generalized estimating equations were used to compare the overall clinical pregnancy rate and cumulative live birth rate between the PCOS group and the control group, and the results showed no statistically significant difference in clinical pregnancy rate(*OR* = 1.083(0.716–1.638), *P* = 0.706) and cumulative live birth rate(*OR* = 0.742(0.314–1.756), *P* = 0.498) between the two groups.

**Table 3:** Pregnancy outcomes between groups

Variables	Control	PCOS	*P* value
ET			
Clinical pregnancy rate	43/117(36.8%)	24/62(38.7%)	0.797
Live birth rate	34/117(29.1%)	19/62(30.6%)	0.825
Full-term birth	27/43 (62.8%)	13/24 (54.2%)	
Preterm birth	7/43 (16.3%)	6/24 (25.0%)	
Ectopic pregnancy	1/43 (2.3% )	2/24 (8.3%)	
Miscarriage	8/43 (18.6%)	3/24 (12.5%)	
FET			
Clinical pregnancy rate	18/66(27.3%)	74/217(34.1%)	0.300
Live birth rate	14/66(21.2%)	63/217(29.0%)	0.211
Full-term birth	11/18 (61.1%)	50/74 (67.6%)	
Preterm birth	3/18 (16.7%)	13/74 (17.6%)	
Ectopic pregnancy	1/18 (5.6%)	1/74 (1.4% )	
Miscarriage	3/18 (16.7%)	10/74 (13.5%)	

Values are described as number/total number ( percentage ). In the ET, the denominator of clinical pregnancy rate and live birth rate was not only the number of patients with fresh embryo transplantation in each group, but also the number of ET cycles in each group; the denominator of full-term birth, preterm birth, ectopic pregnancy and miscarriage is the number of patients with clinical pregnancies in each group, which is also the number of ET cycles. In the FET, the denominator of clinical pregnancy rate and live birth rate was the total number of FET cycles in each group; the denominator of full-term birth, preterm birth, ectopic pregnancy and miscarriage is the number of clinical pregnancy cycles in each group

**Table 4:** Modified Poisson regression for live birth

Groups	Variables	*RR (95%CI)*	*P* value
Control	ET		
	Age	0.864(0.761–0.980)	0.023
	FET		
	Age	0.714(0.522–0.975)	0.034
	Infertility duration (year)	1.181(1.007–1.384)	0.041
PCOS	ET		
	NO		
	FET		
	NO		

**Table 5:** Generalized estimating equations for two groups

Variables	*OR (95%CI)*	*P* value
Clinical pregnancy rate	1.083(0.716–1.638)	0.706
Live birth rate	0.742(0.314–1.756)	0.498

This analysis includes both ET cycles and FET cycles and is an overall comparison of the results of all cycles

## Discussion

In China, with the implementation of the two- and three-child policy, more and more advanced reproductive age women are seeking fertility help. The research on the fertility of advanced reproductive age women has gradually become the focus of public concern and the focus of clinical research. At present, there are few studies on the fertility of advanced reproductive age patients with PCOS, and there is some controversy. Some researchers believe that compared with normal controls, advanced reproductive age patients with PCOS have slower follicle aging, slower decline in ovarian reserve, longer reproductive window than normal women [20, 21], and higher cumulative live birth rate with the help of reproductive technology. However, some researchers believe that advanced reproductive age patients with PCOS do not have an advantage in terms of cumulative live birth rate, and the reproductive window is not prolonged compared with normal advanced reproductive age people.

Obesity may lead to infertility by affecting women’s menstrual cycle, oocytes development, oocytes excretion, and aggravating inflammation [22–25]. The effect of obesity on the outcomes of assisted reproduction is still controversial. Most studies suggest that obesity has a negative impact on clinical outcomes [26, 27], first, obesity can lead to a significant decrease in the number and quality of oocytes [28, 29]; secondly, obesity can lead to a decrease in the patient’s endometrial receptivity, which in turn affects the embryo implantation rate and clinical pregnancy rate [30]; finally, obese individuals are at higher risk of developing gestational hypertension and gestational diabetes, and pregnant women are at increased risk of miscarriage and preterm birth [31, 32]. Another part of the study concluded that obesity does not affect clinical pregnancy outcomes [28, 33]. While 50–70% of PCOS patients showed signs of obesity [34], and had a higher BMI, which was consistent with our findings [(22.30 ± 2.31) kg/m^2^ vs. (25.52 ± 3.38) kg/m^2^, *P* < 0.001]。The influence of obesity on the internal environment and embryos quality of patients may be one of the reasons why the number of embryos in PCOS patients is more, but the clinical pregnancy rate and live birth rate are roughly equal to those in the fallopian tube control group.

The results of our study showed that there was a statistically significant difference in the overall cycle type between the two groups (*X*^*2*^ = 81.018, *P* < 0.001), the PCOS group was dominated by FET cycles (77.8%), and the control group was dominated by ET cycles (63.9%). The results were basically consistent with the actual clinical situation. PCOS patients are a common high-response ovarian population, and they are abnormally sensitive to Gn stimulation, which is a risk factor for OHSS. In order to prevent the occurrence of OHSS as much as possible and reduce the harm of OHSS to patients, after a comprehensive evaluation of patients who use Gn for ovulation induction, especially those with high risk factors, most patients with PCOS choose the strategy of freezing embryos, followed by FET. Freezing all embryos is the most effective measure in the current OHSS prevention strategy, and the clinical pregnancy rates of FET cycles and ET cycles are basically equivalent [35]. Polycystic ovary is one of the typical features of PCOS. The number of oocytes retrieved in the PCOS group was significantly higher than that in the control group (*z*=-8.985, *P* < 0.001), which was consistent with the results of previous studies[16, 17]. Moreover, our study also demonstrated that in assisted reproduction treatment, there was a significant correlation between the number of oocytes retrieved, the number of normal fertilization, the number of available embryos, and the number of high-quality embryos; There is a significant advantage in the number of embryos available and high-quality embryos.

In terms of pregnancy outcomes, our results showed that in ET cycle, there was a statistically significant difference between the two groups in the number of embryos transferred [2.0 (1.0–2.0) vs. 2.0 (2.0–2.0), *P* = 0.041], the number of embryos transferred in the PCOS group was more than that in the control group, the clinical pregnancy rate between the two groups [24/62 (38.7%) vs. 43/117 (36.8%), *P* = 0.7970] and the live birth rate [19/62 (30.6%) vs. 34/117(29.1%), *P* = 0.825] in general agreement, the difference is not statistically significant, contrary to the findings of Mai Z [16]. During the FET cycle, the number of embryos transferred between the two groups was roughly the same [2.0 (2.0–2.0) vs. 2.0 (2.0–2.0), *P* = 0.083], which was consistent with the findings of Mai Z [16]; however, our results showed that the clinical pregnancy rate between the two groups [74/217 (34.1%) vs. 18/66 (27.3%), *P* = 0.300] and the live birth rate [63/217 (29.0%) vs. 14/66 (21.2%), *P* = 0.211] were generally consistent, and the difference was not statistically significant, contrary to the findings of Mai Z [16].

In addition, in the modified poisson regression analysis of the factors affecting the live birth rate, we found age ( ET : RR = 0.864, 95% IC 0.761–0.980, *P* = 0.023 ; FET : RR = 0.714,95% IC 0.522–0.975, *P* = 0.034 ) had an important influence on the live birth rate of ET and FET cycles in patients with simple tubal factor infertility. The younger the age, the higher the live birth rate ; the duration of infertility ( RR = 1.181,95% IC 1.007–1.384, *P* = 0.041 ) only had an important effect on the live birth rate of patients with simple tubal factor infertility in the FET cycle. With the increase of infertility years, the live birth rate gradually decreased. However, in the advanced reproductive age PCOS group, there were no significant influencing factors for either ET or FET cycles. This may be due to the complexity of the condition in patients with PCOS infertility and the fact that the factors influencing live birth rates are not limited to age, embryo and years of infertility. Previous studies have summarised the causes of reduced reproductive potential in PCOS and found that reduced reproductive potential may be the result of a combination of co-morbidities such as altered endometrial function, embryonic, pregnancy complications, oocytes and obesity [12].

Compared with the ovaries of the normal population, more follicles are stored in the ovaries of PCOS patients [9], and the biomarkers of high serum AMH and high AFC are stable, even after the age of 35 years. [10, 16, 36]. Abundant oocytes are a prerequisite for having sufficient available embryos, and also provide a material basis for the improvement of clinical pregnancy rate and cumulative live birth rate. In the latest study, the results of Guan Y [17] et al. suggest that advanced reproductive age patients with PCOS and advanced reproductive age patients with infertility due to tubal factors alone have similar cumulative live birth rates, anti-mullerian hormone (AMH), age and oocyte count play a very important role in the cumulative live birth rate of patients. Kalra et al. believed that before the age of 40, patients with PCOS had a 20–30% advantage in the live birth rate, pregnancy rate and number of oocytes compared with infertile patients with tubal factors, and after the age of 40, the advantage disappeared, with no significant difference between the two groups [37]. Li et al. compared cumulative live birth rates over a two-year period in BMI-matched 35-year-old patients with tubal factor alone and PCOS treated with assisted reproductive technology and showed that PCOS patients had a higher cumulative live birth rate, and the number of transferable embryos and the number of antral follicles were strong independent predictors of cumulative live birth rate [16]. When grouped according to age, the results of Hwang et al. showed that the pregnancy and live birth rates in PCOS patients remained stable until the age of 38, whereas the pregnancy and live birth rates significantly decreased in the control of tubal factors with age increased [38] ,while Mellembakken et al. considered that the pregnancy rate and live birth rate of PCOS patients were stable between 22 and 41 years of age [39]. The results of this study are different from the previous main research results of Mai Z[16], but are generally consistent with the research conclusions of Guan Y[17], compared with the matched advanced reproductive age patients with PCOS and infertility patients with simple fallopian tube factors, there is no significant advantage in reproductive ability, and the reproductive ability of the two is similar. The differences between the results of our study and previous studies may be caused by the following three reasons. First, the inclusion criteria of the study population were inconsistent. Mai Z [16] unified the age of the study population as 35 years, and the BMI and age were matched between the two groups. However, in actual clinical practice, patients with PCOS generally show signs of obesity and even overweight, and more and more studies have shown that obesity has a significant adverse effect on assisted reproductive outcomes [1, 40, 41]. This study was only matched for age, and age was not limited to 35 years, to avoid the confounding bias caused by the matching of weight and age. Second, there is a large difference in sample size. Thirdly, the COS scheme is not unified. This study is a retrospective study, different clinicians have different views and habits in the selection of the program, so there is a certain selection bias, which affects the final IVF/ICSI outcome.

This study expands on previous studies to a certain extent, but there are still some limitations,, mainly in the following four aspects: (1) There is a lack of data on the study of PCOS phenotypes, different phenotypes have different types and degrees of effects on PCOS, adding detailed data on PCOS phenotypes could further improve the completeness of the article. (2) The PSM method can only alleviate systematic differences in observable variables, not in unobservable variation in the variables.3. The study was conducted in the same centre and the sample had some limitations. A multicentre, prospective randomised controlled trial could be conducted to improve the reliability of the findings. 4. This study is retrospective and has limitations, such as the choice of ovulation promotion regimen, where different choices by different doctors can lead to some bias. But the study in the same center has significant advantages in follow-up and other aspects, and a large-scale cohort study with a follow-up rate of nearly 100% can be conducted.

## Electronic supplementary material

Below is the link to the electronic supplementary material.

Supplementary Material 1

Supplementary Material 2

Supplementary Material 3

## Data Availability

All data generated or analysed during this study are included in this published article [and its supplementary information files].
